# Letter to the Editor

**Published:** 2010-08

**Authors:** Aimé Bonny

**Affiliations:** Hôpital Pitié Salpêtrière, Paris, France

Dear Sir

In their article on long-term outcome associated with early repolarisation on electrocardiography, Tikkanen and colleagues (*N Engl J Med* 2009, 24 December)1 refer to the generally admitted definition,2,3 showing a figure with both slurring and notching patterns in subjects who died from arrhythmia. This is the longest-ever published follow-up study on the topic.

It would be useful to have data on the prognostic significance of each type of repolarisation. In our yet-to-be published registry, 4 J-point elevation that was notched rather than a slurred variant appears to be strongly related to the history of transient loss of consciousness in black Africans. However, this finding needs to be studied prospectively.

In addition to the findings that inferior lead localisations and the magnitude of the J-point elevation ≥ 0.1 mV (mostly > 0.2 mV) are stronger predictors of death from cardiac causes or arrhythmia, we want to emphasise the importance of information on the degree of malignancy of each type of early repolarisation on risk-stratification accuracy in subjects with this common pattern in the general population.
